# Effect of flecainide on atrial fibrillatory rate in a large animal model with induced atrial fibrillation

**DOI:** 10.1186/s12872-017-0720-1

**Published:** 2017-12-08

**Authors:** Eva Z. Hesselkilde, Helena Carstensen, Maria M. Haugaard, Jonas Carlson, Steen Pehrson, Thomas Jespersen, Rikke Buhl, Pyotr G. Platonov

**Affiliations:** 1 Department of Veterinary Clinical Sciences, Faculty of Health and Medical Sciences, University of Copenhagen, Højbakkegaard Allé 5, 2630 Taastrup, Denmark; 2 Department of Cardiology, Lund University, 21185 Lund, Sweden; 3 Department of Cardiology, The Heart Centre, University of Copenhagen, Rigshospitalet, Blegdamsvej 9, 2100 Copenhagen, Denmark; 4 Department of Biomedical Sciences, Faculty of Health and Medical Sciences, University of Copenhagen, Blegdamsvej 3, 2200 Copenhagen, Denmark; 5 Arrhythmia Clinic, Skåne University Hospital and Department of Cardiology, Clinical Sciences, Lund University, 21185 Lund, Sweden

**Keywords:** Antiarrhythmic drug, Atrial electrophysiology, Atrial fibrillation, Atrial fibrillatory rate, Animal model, Equine, Flecainide, Horse, Programmed electrical stimulation

## Abstract

**Background:**

Atrial fibrillatory cycle length has been considered one of the indices of atrial electrical remodelling during atrial fibrillation (AF), which can be assessed from surface ECG by computer-assisted calculation of atrial fibrillatory rate (AFR). Horses have been suggested as a bona fide model for AF studies since horses too, develop lone AF, however data on AF characteristics in horses are extremely sparse and non-invasive characterization of AF complexity using surface ECG processing has not been reported.

**Aim:**

The aim was to study characteristics of induced AF and its modification by flecainide.

**Methods:**

The study group consisted on 3 horses with spontaneous persistent AF and 13 with pace-induced AF. Seven horses were treated with saline (control) and eight with flecainide (2 mg/kg). ECGs were analysed using spatiotemporal cancellation of QRST complexes and calculation of AFR from the residual atrial signal.

**Results:**

At AF onset, AFR was 295 ± 52 fibrillations per minute (fpm) in the horses with induced AF treated with flecainide, 269 ± 36 fpm in the control group (ns), and 364 ± 26 fpm in the horses with spontaneous persistent AF (*P* < 0.05 compared to the control group). Flecainide caused a decrease in AFR in all animals and restored sinus rhythm in the animals with induced AF. In the control animals, AFR increased from 269 ± 36 fpm to a plateau of 313 ± 14 fpm before decreasing to 288 ± 28 fpm during the last 10% of the AF episodes preceding spontaneous conversion (*P* < 0.05).

**Conclusion:**

AFR in horses with induced AF resembles AFR in humans with paroxysmal AF. Flecainide caused a rapid decrease in AFR in all horses, further supporting the method to be a non-invasive technique to study the effect of antiarrhythmic compounds.

## Background

Atrial fibrillation (AF) is a self-sustained arrhythmia that progresses over time as a consequence of atrial remodelling. Shortening of the atrial refractory period is considered to be the hallmark of the electrical remodelling, and is closely linked to the atrial fibrillatory cycle length or its reversed measure atrial fibrillatory rate (AFR) expressed in fibrillations per minute (fpm) [1]. As a non-invasive measurement of atrial remodelling, AFR has been repeatedly validated against intracardiac recordings [1, 2] and is expected to become a useful tool for assessment of AF treatment strategies.

Recently, the horse has been proposed as a bona fide model for AF. [3, 4] Like humans, horses develop AF both with and without underlying cardiac diseases, which is believed to be a consequence of their large hearts and high vagal tone. [5] In addition to the naturally occurring cases, AF can be electrically induced in horses, allowing studies of AF remodelling and treatment regimes.

In clinical medicine, flecainide is a commonly used drug to restore sinus rhythm in patients with recent-onset AF. [6] There is an ongoing debate over the effectiveness and safety of flecainide as an antiarrhythmic drug in horses, as the literature shows conflicting results. A study by Ohmura et al. [7] describes flecainide to be safe and efficient in restoring sinus rhythm in an experimental setting, whereas a Belgian group reported disappointing efficacy [8] and fatal side effects. [9] AFR has been reported to decrease after flecainide treatment [3], but whether non-invasive AFR assessment is suitable for monitoring drug effects in horses (based on the processing of surface electrocardiogram (ECG) recordings) is yet to be studied.

## Methods

The aim of this study was to assess AFR behaviour in horses with atrial fibrillation and to study the effects of flecainide on AFR using time-frequency analysis applied to Holter ECGs.

ECGs from 13 Standardbred horses were collected. All horses were electrically stimulated into AF using programmed electrical stimulation from a multipolar catheter placed in the right atrium through the jugular vein. After AF induction the horses were either treated with saline (control group) or flecainide (flecainide group). Continuous modified base-apex ECG was recorded during the entire duration of the experiments. [3] The control group consisted of four mares and three geldings (mean age 9.1 years, range 4–14; mean bodyweight (BW) 497 kg, range 454–555). The flecainide group consisted of five mares and two geldings (mean age 7.4 years, range 4–16; mean BW 474 kg, range 390–552). One horse received both saline and flecainide with a 6-day wash-out period in between.

Following AF induction, a minimum of 15 min continuous AF was required before initiation of treatment with either Flecainide (Tambocor, Meda AS, Allerød, DK; *n* = 7) or isotonic saline (n = 7). Flecainide (2 mg/kg) or saline were infused intravenously (IV) over a period of 10 min. Five horses received three additional IV saline injections (0.0125 ml/kg/min) 20 min apart. After infusion the horses were monitored at rest until either drug induced or spontaneous cardioversion occurred. In order to minimize the risk of untimely spontaneous cardioversion in horses scheduled for saline infusion, they were required to have an AF episode of at least 1-h duration to be included in the study.

Additionally, three horses with spontaneous AF documented with long-term continuous ECG registration were included in the study (two Standardbreds, one Warmblood), one mare and two geldings (mean age 8 years, range 7–10; mean BW 514 kg, range 464–548). Horse A had successfully been treated for AF with Quinidine Sulfate twice within recent years. The AF episode included in this paper had lasted more than 3 weeks prior to flecainide treatment as described above. The AF duration in horse B was also known to be over 3 weeks, whereas it was only possible to determine that the AF duration in horse C was a minimum of 12 h, as AF was an incidental finding and the horse was brought straight to the clinic. There were no records of previous AF episodes for horse B or C and no flecainide treatment were attempted.

### ECG recordings and signal processing

ECGs were recorded as a modified base-apex ECG. All recordings for the induced horses included AF onset, saline or flecainide infusion and termination of AF, while the spontaneous persistent horses had minimum of 60 min ECG recorded and analysed. All ECG recordings were manually reviewed for quality assessment and the presence of AF in the Televet system (Kruuse, Langeskov, DK) or Labchart software (LabChart 7 Pro, ADInstruments, Oxford, UK). ECG analysis including QRST cancellation and AFR calculation was performed using the Cardiolund AFR Tracker software (cardiolund.com). [10] AFR values where less than 10% of the signal could be used for automated analysis were discarded as missing values.

### Statistical analysis

Data are presented as mean ± SD or mean ± SEM as appropriate. To account for different AF duration when studying AFR behaviour in the control horses, the median of the AFR values in the 10th, 45-55th and 90-100th percentiles of AF duration in the individual horses were calculated, and a Friedman’s one-way ANOVA was performed, followed by Dunnett’s post hoc test. AFR values were compared between groups using a Mann-Whitney unpaired t-test, while a comparison of AFR values before and after treatment was performed using Wilcoxon matched pairs t-test where the mean of the last 10 min prior to treatment was compared to the last AFR value before cardioversion (flecainide group) or mean of the following 10 min after injection start (control group). The threshold for statistical significance was *P* < 0.05 throughout the study.

## Results

The quality of the ECG recordings was generally good and few segments (5.3%) of the ECGs were discarded due to excessive levels of noise.

For the AF-induced horses, the mean AF duration in the control group was 205 min (range 152–273) and 26 min (range 16–36) in the flecainide group.

### AFR dynamics in control horses during induced AF

**Fig. 1:**
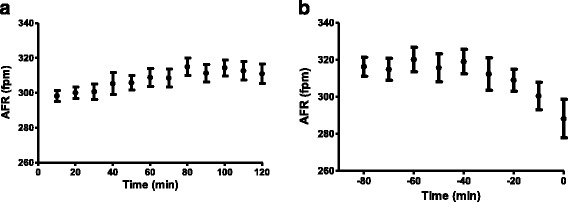
AFR in control horses at AF onset (**a**) and before spontaneous cardioversion (**b**). **a** illustrates an increase in AFR in the first 70 min before reaching a plateau. **b** illustrates the gradual decrease in AFR 30 min before restoration of sinus rhythm at time = 0. Values are presented as mean ± SEM, *n* = 7

AFR at initiation of AF was 269 ± 36 fpm (range 208–317). AFR significantly increased in all horses from the 10th percentile (299 ± 10 fpm) to the 45-55th percentile (313 ± 14 fpm; *P* < 0.05). Before spontaneous cardioversion, AFR significantly decreased again at the 90-100th percentile (302 ± 19 fpm; P < 0.05). Table 1 describes the AFR and time at the different stages. The difference between percentiles suggests a plateau or “steady state” during which AFR was stable. Studying the individual trends, the time to the plateau was 40 to 70 min in six of the horses, while AFR in one horse continued to increase for 140 min. The average time to plateau was 68 ± 33 min, as illustrated in Fig. 1. All horses showed a decrease in AFR during the last 30 ± 17 min prior to spontaneous cardioversion.

**Table 1:** AFR behaviour in the control horses

	AFR (fpm)	Time (minutes)
Initiation of AF	269 ± 36^†^	1
0-10th percentile	299 ± 10^†^	21 ± 4.9
45-55th percentile	313 ± 14	92 ± 22–112 ± 27
90-100th percentile	302 ± 19^†^	183 ± 44–204 ± 49
Last value prior to termination	288 ± 28^†^	205 ± 50

AFR (fpm) and time (minutes) described at initiation of AF, the first, middle and last 10% of the AF episodes, and the last value before termination. All values are presented as mean ± SD

^†^Differs significantly from the 45-55th percentiles (*P* < 0.05)

### The effect of flecainide on AFR during induced AF

**Fig. 2:**
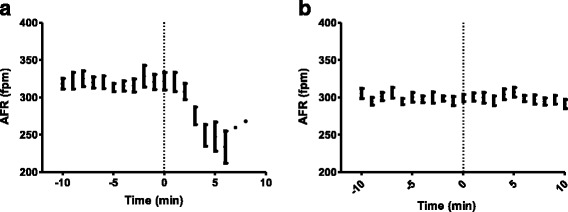
AFR 10 min before and after treatment was initiated (at time = 0). Data are presented as mean ± SEM. **a** represents the horses treated with flecainide. Note the rapid change in AFR as a consequence of flecainide administration. **b** represents the horses treated with saline

At AF onset, AFR was 295 ± 52 fpm in the flecainide group, which was non-significant compared to the control group (*P* > 0.05). Flecainide restored sinus rhythm in all horses with induced AF. The duration of AF episodes before treatment was on average 22 ± 5 min, and time to cardioversion after flecainide infusion started was 4.3 ± 2.5 min. AFR decreased immediately after the initiation of flecainide infusion (*P* < 0.05). In contrast, AFR in the control group remained unchanged after saline infusion (*P* > 0.05). Figure 2 illustrates the behaviour of AFR after infusion with flecainide (2a) and saline (2b).

### AFR in horses with spontaneous persistent AF

**Fig. 3:**
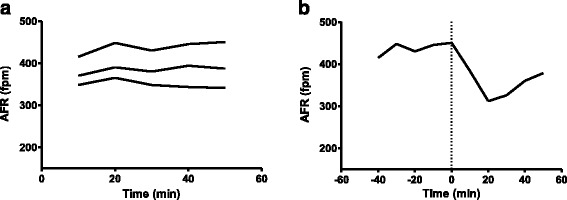
AFR in horses with spontaneous persistent AF. Data are presented as 10-min average. **a** illustrates a stable AFR without drug intervention (*n* = 3) and **b** illustrates the attempt to terminate AF with flecainide (injection time = 0, *n* = 1). Note the significant decrease in AFR after flecainide infusion, followed by an increase as the flecainide failed to restore sinus rhythm

The horses with spontaneous persistent AF presented with a significantly higher AFR compared to the horses with electrically induced AF (*P* < 0.05; mean of the first AFR value recorded 364 ± 26) and remained stable in the absence of drug intervention (Fig. 3a). Sinus rhythm was not restored in response to flecainide administration in horse A, despite a decrease in AFR of more than 100 fpm 10 min after infusion (Fig. 3b).

## Discussion

### Main findings

In the present study, we found that AFR in horses closely resembles AFR in humans, both in terms of rate, behaviour over time, and response to drug injection. We found an AFR range of 250–350 fpm in seven horses with induced AF, which is well correlated with clinical observations where patients with induced AF had a frequency of 336 ± 78 beats/min, [2] and patients with paroxysmal AF reached a rate of 326 ± 39 fpm 3 h after onset. [11] Two independent clinical studies have suggested cut-off values of less than 350 fpm [12] and 355 fpm [13] for predicting spontaneous cardioversion. As all AF-induced horses receiving saline in this study, had AFR below this threshold and cardioverted spontaneously, it seems that these cut-off values may also apply to horses. Atrial remodelling will advance as AF accelerates as a consequence of AF itself. [14] As AFR can be used to assess atrial remodelling, it is reasonable to expect that AFR will evolve with increased AF duration. In agreement, we found the three horses with spontaneous persistent AF to have a mean of the first AFR value recorded at rest of 364 ± 26, which is considerably higher than the short-term induced AF and also in agreement with human AF studies, in which patients with AF episodes longer than 3 months had a frequency of 402 ± 78 beats/min. [2]

### AFR dynamics in control horses

In the present study, we found an increase in AFR from AF onset, and six out of seven horses had reached a plateau after 70 min. Using similar methodology, Bollmann et al. [15] and Petrutiu et al. [16] reported time to a plateau being 4–5 min in patients with short AF episodes that lasted several minutes. Platonov et al. [11] observed an increase in AFR during the first 3 to 4 h in a study where one patient had 8.5 h of AF, and the remaining three patients had AF episodes of more than 48 h. The mean AF duration in our control horses was 3.4 h, suggesting that time to reach a plateau may depend on the duration of the AF episode, since both the length of the episodes and AFR are likely to reflect the degree of the atrial remodelling. This is further supported by the observations that no change in AFR was found when the duration of AF episodes was less than 15 min. [15] This study also indicates that horses with longer AF duration reached the plateau later. However, it is important to note that AF was induced in these horses, and that the sample size was rather small.

Before termination of AF, we observed a pronounced decrease in AFR. As with the initial AFR increase, the literature presents some inconsistencies on this matter – Bollmann et al. [2] observed no changes in AFR towards termination using a 10-s time frame, while Petrutiu et al. [16] observed an abrupt decrease when AFR was studied on a second-to-second basis. In contrast, Platonov and colleagues [11] observed a gradual decrease 1 h before spontaneous cardioversion in one patient, while Fujiki [17] demonstrated a slowing of AFR 10 min prior to termination in patients that cardioverted in the morning. It is most likely that the different observations were due to different AF durations, suggesting that short-term AF episodes terminate more quickly with little or no change in AFR, while longer AF episodes have a gradual AFR decrease before spontaneous termination.

### The effect of flecainide on AFR

Flecainide is a class I antiarrhythmic drug that slows down atrial fibrillatory process in horses [3, 8] and in humans. [18] The present study showed a clear effect of flecainide on AFR retrieved from surface ECGs, as AFR dropped immediately after infusion started. The same effect was seen when 18 patients with AF were treated with oral flecainide, which resulted in a frequency drop of 30%. [19] Patients with low frequency atrial fibrillation were more likely to cardiovert spontaneously or in response to treatment than patients with high frequency fibrillation. [15] Our study shows that this may also apply to horses, as flecainide terminated AF in the induced horses that had a significantly slower rate than those with spontaneous persistent AF. Flecainide reduced AFR by 100 fpm to a level similar to the induced horses, yet this was not sufficient, as AFR started to increase again before cardioversion, leaving the horse in AF. Likewise, Bollmann et al. [15] showed that AF frequency decreases in response to flecainide treatment (*P* < 0.001) without restoring sinus rhythm. Several other studies have investigated the effect of a number of antiarrhythmic drugs, [20] all shown to reduce AFR. This supports AFR as a non-invasive method of tracking the effect of antiarrhythmic compounds in order to understand the effect on AF in humans and now also in horses.

## Conclusion

To our knowledge, this is the first analysis of the non-invasive characterization of AF in horses. AFR values and their evolution during induced AF in horses resemble AFR dynamics in humans with paroxysmal AF, and horses with spontaneous persistent AF demonstrate an increase in AFR similar to patients with long-lasting AF. Flecainide caused a rapid decrease in AFR, suggesting that AFR measurement is a non-invasive method to monitor the effects of antiarrhythmic compounds in AF and supporting the validity of the horse as a model for human AF.

## Abbreviations

AF: Atrial fibrillation
AFR: Atrial fibrillatory rate
BW: Body weight
ECG: Electrocardiogram
Fpm: Fibrillations per minute
IV: Intravenous
